# Influence on Sample Determination for Deep Learning Electromagnetic Tomography

**DOI:** 10.3390/s24082452

**Published:** 2024-04-11

**Authors:** Pengfei Zhao, Ze Liu

**Affiliations:** The School of Automation and Intelligence, Beijing Jiaotong University, Beijing 100044, China; 16111044@bjtu.edu.cn

**Keywords:** electromagnetic tomography, deep learning, sample determination, image reconstruction

## Abstract

Deep learning (DL) has been frequently applied in the image reconstruction of electromagnetic tomography (EMT) in recent years. It offers the potential to achieve higher-quality image reconstruction. Among these, research on samples is relatively scarce. Samples are the cornerstone for both large and small models, which is easy to ignore. In this paper, a deep learning electromagnetic tomography (DL-EMT) model with nine elements is established. Complete simulation and experimental samples are obtained based on this model. On the sample sets, the reconstruction quality is observed by adjusting the size and configuration of the training set. The Mann–Whitney U test shows that beyond a certain point, the addition of more samples to the training data fed into the deep learning network does not result in an obvious improvement statistically in the quality of the reconstructed images. This paper proposes a CC-building method for optimizing a sample set. This method is based on the Pearson correlation coefficient calculation, aiming to establish a more effective sample base for DL-EMT image reconstruction. The statistical analysis shows that the CC-building method can significantly improve the image reconstruction effect in a small and moderate sample size. This method is also validated by experiments.

## 1. Introduction

Electromagnetic tomography (EMT) could reconstruct the distribution of the unknown object without invading the interior, also known as magnetic induction tomography (MIT) in research. Other ET technologies include electrical impedance tomography (EIT), electrical capacitance tomography (ECT), and electrical resistance tomography (ERT) [1]. Compared to these, EMT has characteristics sensitive to both conductivity and permeability. EMT has been studied and applied in the fields of industrial process tomography [2,3,4,5] and biological tissue imaging [6,7].

### 1.1. Background

Although EMT technology has been extensively studied, there is still room for breakthroughs in EMT technology to achieve faster and more accurate measurements. From the original perspective, the mathematical principle of computed tomography (CT) and EMT is the Radon transform [8]. Unlike CT, EMT’s physical field is a soft field, which implies that the process of image reconstruction is ill-posed. This also indicates that numerous mature tomography algorithms cannot be used for EMT directly. Considering the above situation, the prior information utilized in the classical EMT algorithm may not be sufficient. Prior information refers to information that has already been obtained before actual measurement. Facing an abundance of prior information, the development of machine learning, especially deep learning, provides a natural method to solve this problem. It can learn the inherent laws and representation levels from a large amount of sample data [9], which has already achieved remarkable success in the field of natural language processing [10] and image recognition [11]. Furthermore, the advent of innovative graphical modeling techniques has expanded the horizon of deep learning into dynamic system analysis, offering nuanced insights into system behaviors through graphical representations [12,13].

In particular, due to the high commonalities and similarities between DL-EMT and deep learning–electrical tomography (DL-ET) research, much of the literature and explanations in this chapter also utilize relevant explanations from DL-ET. As the predecessor of deep learning, the research of neural networks in ET began early. In 1994, Nooralahiyan, Hoyle, and Bailey used a single-layer multioutput network (they even named it SLMON) to image different sets of simulated patterns. The training sets include data for an empty pipe (filled with gas), a full pipe (filled with oil), and 37 sets of data consisting of the smallest identifiable gas components (bubbles) in oil [14]. Since 2017, Li et al. have applied the stacked autoencoder (SAE) and a logistic regression (LR) layer to constitute a four-layer DNN model [15]. Our previous work constructs a stack sparse autoencoder (SSAE) with a Radial basis function (RBF) network and optimized fully connected (Optimized FC) network to achieve the image reconstruction of EMT. After adding 7% noise, the image reconstruction results are relatively excellent [16]. Zheng J. et al. use the fully connected network in deep learning to compensate the reconstruction image of the classical Linear Back Projection (LBP) algorithm [17]. Li et al. combine the mathematical structure of the Landweber image reconstruction algorithm with deep learning to form a Landweber iterative reconstruction network, and the generalization ability was improved [18]. This also shows that the research on tomographic image reconstruction has begun to enter another stage. Instead of simply using successful networks in other fields, Li has begun to develop networks combined with tomographic specificity.

In recent research, graph convolutional networks (GCNs) have been employed to improve the resolution and quality of ECT image reconstruction. This approach utilizes the ability of GCNs to effectively utilize specific geometrical relationships hidden within unstructured grids that are commonly used to construct 3D ECT images [19]. Ye et al. focus on effectively fusing the measurement information from both tunnel magneto resistance sensor electromagnetic tomography (TMR-EMT) and ERT by directly processing the features of the measurements. This is achieved through a multiscale mixed-attention network model that excels in reconstructing the distribution of gas–liquid–solid phases by extracting both deep and shallow features from the measurement information and applying mixed-attention blocks for simultaneous feature fusion in channel and spatial dimensions. The model demonstrates superior fusion effects and reconstruction quality compared to traditional algorithms, showcasing its robust anti-noise performance and generalization capabilities [20]. Zhang et al. propose a new deep-learning-based method for EMT to simultaneously reconstruct both conductivity and permeability distributions. The methodology involves initially measuring the mutual inductance and magnetic induction intensity in the imaging area, followed by employing the Landweber algorithm to reconstruct initial images of the conductivity and permeability. These initial images are then refined using an improved DeepLabv3 network to achieve high-quality reconstructions with clear boundaries and accurate positions. Experimental results demonstrated the effectiveness of this approach [21].

### 1.2. Problem Statement

**Figure 1:**
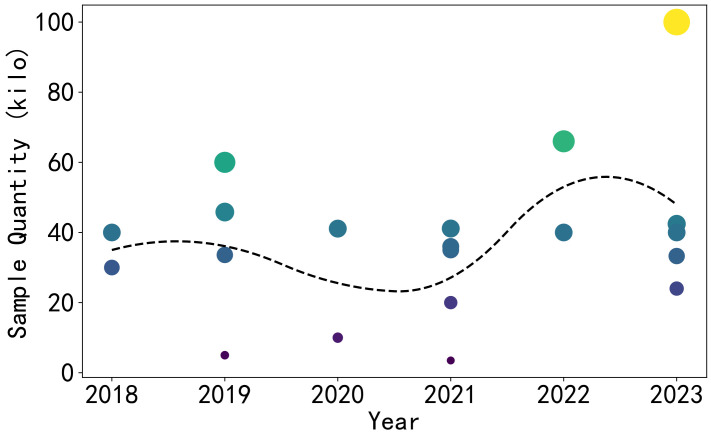
The relationship between the number of samples and the year of publication.

This is the change in sample size used in 21 randomly selected papers on DL-ET in recent years [16,22,23,24,25,26,27,28,29,30,31,32,33,34,35,36,37,38,39,40,41]. In Figure 1, the size of each point represents the size of the sample set used in an article, while different colors are assigned to them for easy distinction. From Figure 1, the number of samples varies from a few thousand to one hundred thousand. Li et al. generated a dataset, a relatively large sample set containing 100,000 data samples through numerical simulation. Each sample includes a normalized capacitance vector and a particle concentration distribution image [28].

As for the types of samples, many scholars reference common flow patterns in multiphase flow. Wu et al. used simulation to construct a large number of flow pattern samples, including various types such as single bubble flow, double bubble flow, three bubble flow, four bubble flow, five bubble flow, annular flow, and stratified flow. Each flow pattern consists of 5000 samples, totaling 35,000 sets [39]. Luo et al. also built a simulated dataset. This dataset contains 33,280 samples, with one bubble comprising 8569 samples, two bubbles 19,475 samples, and three bubbles 5236 samples. The bubble diameters range from 5 mm to 25 mm, representing the dispersed phase in the fluid medium [41]. Although several datasets aim to advance the development of ECT, the Benchmark Dataset established by Zheng et al. occupies a unique position in this field. The Benchmark Dataset includes a 2D model and 3D model. Within the 2D model, there are 40,000 samples, each including a normalized dielectric constant distribution vector (3228 dimensions) and a corresponding normalized capacitance vector (28 dimensions under an eight-electrode ECT sensor). These samples are based on various flow patterns, such as annular, stratified, single-bar, and two-bar. Moreover, each flow pattern is represented by 10,000 samples. The rationale behind designing these four flow patterns is that they are typical two-phase flow types commonly encountered in the industrial sector, and other complex flow types can be considered as combinations of these flows. The 3D model portion includes capacitance vectors corresponding to 80 different operating conditions, including capacitance vectors for full and empty tubes used for calibration, two sensitivity matrices for the 8-electrode and 12-electrode models, and twelve normalized dielectric constant distribution vectors. Based on the above description, the design of this Benchmark Dataset can provide a good benchmark platform for evaluation.

Regarding the principles of sample generation, our previous work designed a total of 30,000 samples of two types, with 15,000 of each type. The first sample type includes 1 to 4 randomly distributed small objects to learn the distribution characteristics of small and multiple objects. The second sample type features a single large object with a random shape and position, occupying 25–40% of the object field area, intended for learning the contour distribution of large objects. The principle of sample generation was first to delineate the primary sample type and then generate them randomly [16]. Through a literature review, it is discovered that many studies essentially adhere to the following design principles: Firstly, the sample type is selected. Then, specific samples generated within a particular sample type are usually produced randomly. Tan et al. designed 41,122 samples for use in ERT, and the design principle was based on professional experience. Each sample comprises 208 boundary voltage measurements and an associated conductivity distribution of 812 elements. Inclusions are set to simulate gases with low conductivity. The design principles are based on professional experience [22]. In Anna et al.’s research, the dataset comprised signals containing one and two perturbation objects, totaling 66,000 examples. The signals were stored as one-dimensional vectors with a dimension of 1236. This dataset was meticulously designed [30]. The design of the samples in these studies is usually based on experience, with few research efforts optimizing the number and composition of samples based on data and algorithms. Such research is necessary because experience is highly subjective, and analyzing and optimizing samples based on data and algorithms can serve as an effective supplement and correction to experience.

Additionally, it has been found that sample sets with a larger volume of data, typically over 10,000 samples, are almost exclusively generated through simulations. Some samples are produced experimentally, but they are generally used for testing or added to the training set in small quantities to enhance the generalization capability. Overall, we found little attention and research dedicated to samples in EMT-DL and ET-DL.

### 1.3. Research Objectives and Contributions

In the vast majority of cases, it is impossible to obtain the complete set of samples. Only a portion of the samples can be obtained under certain conditions within a certain period. For the need to generate samples, although the process of creating samples involves additional costs, the fact that the samples are self-produced also provides more excellent selectivity. At this juncture in an era, we would like to use a small, basic, but complete model to describe some issues, making necessary preparations for future Large-Scale Models in EMT and even ET.

This research introduces innovative methodologies in DL-EMT, focusing on the role of sample determination in enhancing image reconstruction accuracy. By establishing a nine-element EMT model and generating a comprehensive set of both simulated and experimental samples, this study explores the marginal effects of increasing sample size on image quality. We propose a CC-building method for optimizing the sample set. The findings reveal that while enlarging the sample set, the benefits decrease. Moreover, the CC-building method demonstrates its effectiveness in achieving superior image reconstruction with fewer samples, marking a significant step forward in the efficient application of deep learning in EMT. These contributions not only advance the field of electromagnetic tomography but also offer valuable insights for broader applications in deep-learning-based imaging technologies.

Section 1 describes the background of the research on sample determination. Section 2 describes the theory and methods of EMT, the nine-element model, and the network. Section 3 describes the simulation results and proposes the CC-building method and the sample determination pyramid. Section 4 describes the experimental verification of Section 3. Section 5 describes the conclusion and suggestions for sample determination.

## 2. Method

This section mainly introduces the fundamental theories of EMT, as well as the EMT model and deep learning network used in this paper.

### 2.1. Theory of EMT

EMT mainly studies the relationship between the internal distribution of conductivity σ, magnetic permeability μ, and boundary detection values. According to Maxwell’s equations, in the absence of free charges in space, it can be obtained that

(1) ∇·D=0

When the excitation frequency is low $\left(\omega <10^{7}\right)$, the influence of the displacement current could be ignored:

(2) ∇×H=σ(x,y)E

Introduce the vector magnetic potential A to satisfy

(3) ∇×A=B

Input Equation (3), $H=\frac{B}{\mu }$, into Equation (2) and obtain

(4) $$\nabla \times \left(\frac{\nabla \times A}{\mu }\right)=\sigma \left(x,y\right)E$$

By vector identity $\nabla \times \nabla \times A=\nabla \left(\nabla \cdot A\right)-\nabla^{2}A$ and Coulomb gauge ∇·A=0 , obtain

(5) $$\nabla^{2}A=-\mu \sigma \left(x,y\right)E$$

Input Equation (3) into ∇×E=−jωB and obtain

(6) ∇×(E+jωA)=0

Introduce the scalar potential φ and obtain

(7) E=−∇φ−jωA

Therefore, we could obtain

(8) $$\nabla^{2}A=j\omega \mu \sigma \left(x,y\right)A$$

The above equation illustrates that in EMT, the presence of conductivity and permeability can affect the vector magnetic potential in space, which in turn affects the detection signal of edge sensors. This provides theoretical support for electromagnetic tomography imaging technology.

### 2.2. Model Construction

In general, the region of interest (ROI) in EMT refers to all areas to be detected, where the internal division ranges from tens to thousands of elements. In this context, elements refer to specific subdivision units. Due to the realities of the situation, the areas that need to be detected in EMT are often complex. Therefore, the number of subdivisions within the detection area needs to be as large as possible; that is, the subdivisions should be as fine as possible. Generally, this process is time consuming, but in some situations, smaller subdivisions are more helpful in discovering some universal patterns.

**Figure 2:**
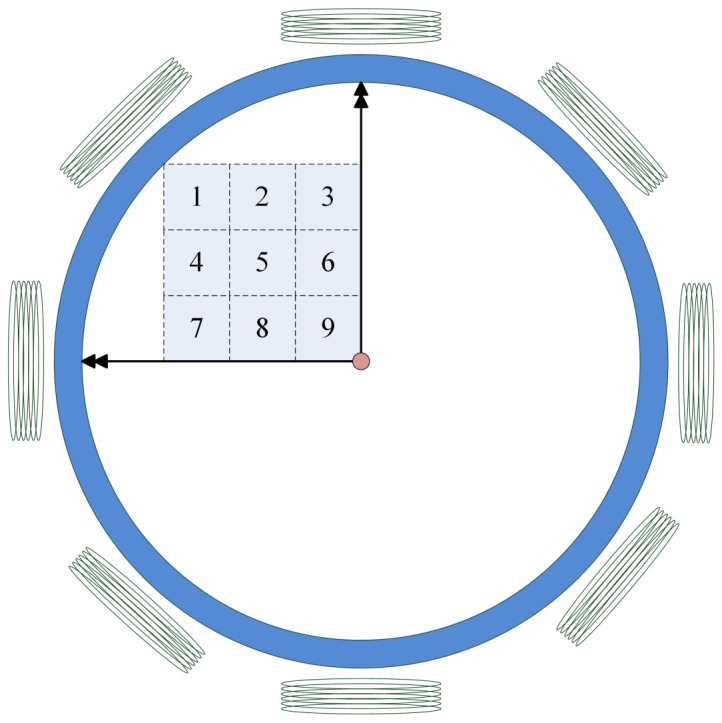
The 9-element EMT model.

This paper constructs a 9-element basic EMT model as in Figure 2. Its ROI has only 9 elements, and the amount of all possible internal distributions is $2^{9}=512$, which can all be obtained. Hence, the sample set of this model is complete. The 9 elements are distributed in the upper-left area of the entire measured area, covering the areas from the edge (more sensitive) to the center (less sensitive). Each element occupies 1.8 % of the cross-sectional area of the entire circle.

The advantage of this model is that it exhibits rotational symmetry. This preserves the scalability of the model, allowing for a 4-fold expansion of the sample when each coil has isotropic consistency. Drawing on previous research—specifically, the study by Han et al.—it has been demonstrated that data from different ROIs (regions of interest) can be unified within a single neural network [42]. Therefore, conducting preliminary small-scale experiments becomes more direct and can better guide subsequent research efforts.

The model in this paper is the basic 8-coil circular model in EMT. Given that the objective is to conduct a comparison among multiple batches of samples, the choice of network has a minimal impact on the outcomes. The network selected for this study is the Fully Connected Neural Network (FCNN), one of the most elementary forms of artificial neural networks. Within this network, every neuron in a fully connected layer is connected to all neurons in the preceding layer, where the input of the whole network is detection signals. The output is the gray vector of the desired image. The output of each neuron is

(9) $$x_{i}^{n+1}=h\left(f_{BN}\left(b_{i}^{n}+\sum_{j=1}^{m}W_{ij}^{n}x_{j}\right)\right)$$

In all subsequent tests in this paper, the hyperparameters of each test are kept constant to ensure that the results are influenced solely by variations in the sample.

## 3. Sample Configuration and Simulation

The network is constructed and implemented in PyCharm based on the TensorFlow environment. Tensorflow is widely used in the implementation of various machine learning algorithms. Because each sample of DL-EMT requires its own design and production, this paper mainly explores two issues: the impact of sample size on the results and what kind of sample combination constitutes a more optimal grouping.

### 3.1. Impact of Sample Size

**Figure 3:**
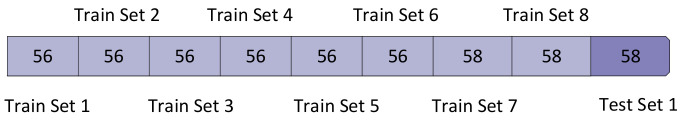
The distribution of sample set.

In the simulation and experimental measurement in this paper, the same set is selected as the test set, which includes 58 samples. The remaining samples are selected as train set 1, train set 2, ..., and train set 8 in incremental order of 1-norm. The number of samples is shown in Figure 3. This allocation method is referred to as ’Raw-1’.

Then, a four-layer FCNN is built. According to the Reciprocity Theorem, the input is a $\frac{n\times (n-1)}{2}=28$-dimensional detection signal. The output is a nine-dimensional gray distribution. Because the order of magnitude of the input and output is minor, in order to prevent over-fitting, the number of neurons per layer is 200. Since the focus is solely on the impact of sample determination on the results, it is necessary to ensure consistency in the hyperparameters. Hence, no validation set was established.

After the network is established, the implementation of the image reconstruction process in this paper is implemented in two steps. First, train the network with a pre-determined training set. Second, use the test set to test the results on the trained network. In this study, the loss function employed is the cross-entropy loss function. It could establish associative relationships between various outputs while concurrently expanding the distance between them. Here, H(P,Q) denotes the cross-entropy, which is expressed as follows:

(10) $$H\left(P,Q\right)=-\sum_{i=1}^{n}P\left(x_{i}\right)logQ\left(x_{i}\right)$$

**Figure 4:**
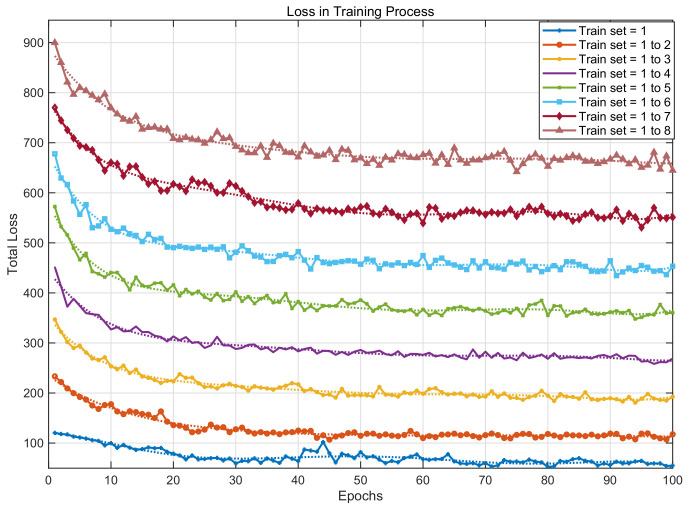
Cross-entropy loss of different train sets.

Then, train set 1 (sample numbers: 58), train set 1 to 2 (sample numbers: 112), ..., train set 1 to 8 (sample numbers: 448) are sequentially utilized as training sets for the network. From Figure 4, it can be seen that as the epochs increase, the cross-entropy loss gradually decreases and converges. A total of 100 epochs is a relatively reasonable setting. Because the ideal value of the total cross-entry H(P,Q) is not zero and will increase with the increase in the train set, the overall curve gradually moves upward.

Based on the eight networks trained with train set 1, train set 1 to 2, ..., train set 1 to 8, the test set is used to test the results on the eight trained networks. For the nine-element model, the training process and image reconstruction speed is quick. If the number of elements increase, the reconstruction speed will decrease along with the calculation complexity increasing. Then, we obtained a total of 58×8 gray-scale images predicted by eight different networks. The evaluation parameters for the reconstructed images are the image correlation coefficient (ICC) and the relative image error (RIE). Generally, reconstructed images with a higher imaging quality have a higher ICC and lower RIE than the ground truth:

(11) $$ICC=\frac{\sum_{i=1}^{N}\left(\hat{g_{i}}-\bar{\hat{g}}\right)\left(g_{i}-\bar{g}\right)}{\sqrt{\sum_{i=1}^{N}{\left(\hat{g_{i}}-\bar{\hat{g}}\right)}^{2}\sum_{i=1}^{N}{\left(g_{i}-\bar{g}\right)}^{2}}}$$

(12) $$RIE=\frac{∥\hat{g}-g∥}{∥g∥}$$

**Figure 5:**
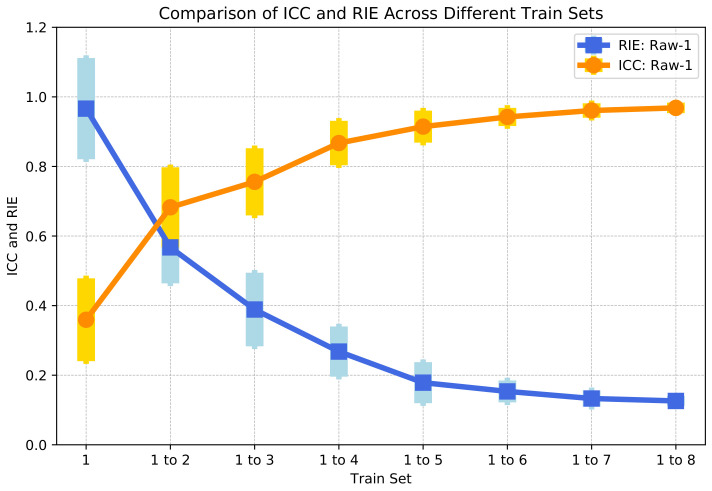
The ICC and RIE of Raw-1.

The mean value and variance of the ICC and RIE of 58 samples in each group are calculated as shown in Figure 5. It can be seen that as the training set increases uniformly, the ICC increases and the RIE decreases. This means that the more samples participate in the training, the higher the correlation between the imaging of the test set and the ground truth, and the smaller the image error is. This is consistent with our subjective perception that the larger the training set, the better the imaging effect is.

However, it could be found that the increasing trend of the ICC and the decreasing trend of the RIE are gradually slowing down. This reflects that there are differences in the improvement in imaging accuracy with the addition of samples based on different sample sizes. This is quite similar to the description of marginal utility in economics. It refers to the phenomenon where there is a continuous increase in a particular input when other inputs remain fixed, resulting in a gradual decrease in the added output or income. That is to say, once the level of increased input surpasses a certain threshold, the output produced by each additional input unit will decline.

The Mann–Whitney U test is introduced to analyze this process from a statistical perspective. The Mann–Whitney U test, also known as the Wilcoxon rank-sum test, is a nonparametric statistical test that is used to compare two independent samples to assess whether their population distributions differ significantly. Given the larger sample size (58 samples) in the calculation of the *p*-value in the Mann–Whitney U, we employed the normal approximation to calculate the *p*-value. The mean $\mu_{U}$ and standard deviation $\sigma_{U}$ of the U distribution were determined using the formulas

(13) $$\mu_{U}=\frac{n_{1}n_{2}}{2}$$

(14) $$\sigma_{U}=\sqrt{\frac{n_{1}n_{2}\left(n_{1}+n_{2}+1\right)}{12}}$$

where $n_{1}$ and $n_{2}$ are the sample sizes of the two groups. The Z-score was calculated by subtracting the mean of *U* from the observed *U* value and then dividing by the standard deviation of *U*:

(15) $$Z=\frac{U-\mu_{U}}{\sigma_{U}}$$

**Figure 6:**
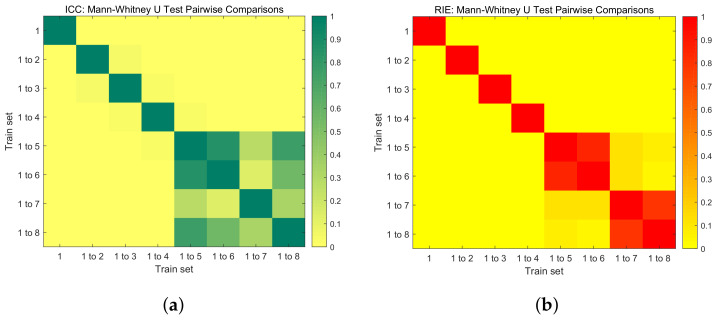
Mann–Whitney U test pairwise comparisons. (**a**) ICC: Mann–Whitney U test. (**b**) RIE: Mann–Whitney U test.

The Z-score was then used to find the corresponding *p*-value from the standard normal distribution. The *p*-value represents the probability of obtaining the observed *U* value (or more extreme) under the null hypothesis that the distributions of the two groups are the same.

p<0.05 is generally considered to indicate a statistically significant difference between two sample groups. Conversely, p⩾0.05 suggests that there is no statistically significant difference between the groups. As illustrated, Figure 6a presents the Mann–Whitney U test of the ICC between pairs of the eight trained networks, while Figure 6b displays the Mann–Whitney U test of the RIE between pairs of the eight trained networks. From these figures, it is evident that starting from ’Train set = 1 to 5’, there is no statistically significant difference in the ICC and RIE for the reconstructed images. Therefore, beyond this point, increasing the number of training sets does not significantly improve the quality of image reconstruction.

Based on these findings, we can draw the following conclusion: it is feasible to continuously increase the sample size for the sake of precision. However, the samples cannot be increased indefinitely because of the sample production cost. Once the sample size reaches a certain level, it needs to be evaluated. If the contribution of adding more samples to the quality of image reconstruction is minimal, then it is not entirely meaningful to continue increasing the sample size. Therefore, according to specific usage requirements, it is sufficient that the precision meets the necessary criteria.

### 3.2. Optimizing Sample Set

**Figure 7:**
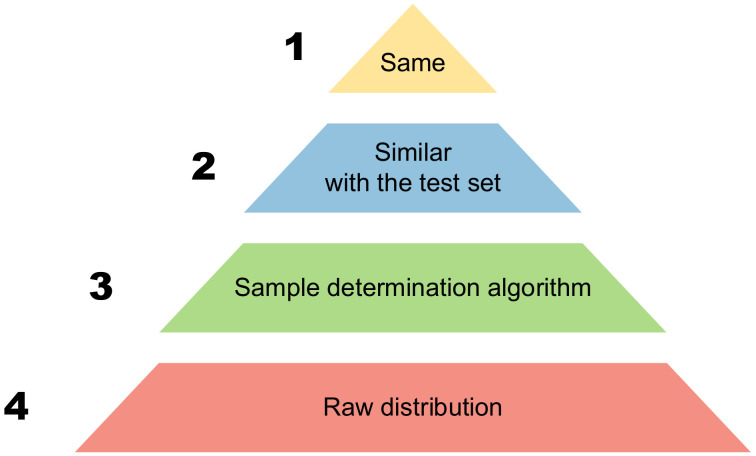
The sample determination pyramid.

To address optimal sample combination with a fixed sample size, we propose a pyramid for sample determination, as shown in Figure 7. The order of the pyramid, from top to bottom, generally has four levels. The fourth level of the pyramid refers to the coarse distribution, which refers to the distribution of samples with simple determination or without determination. The third level of the pyramid is to select the samples to be generated in advance through the sample determination method without knowing the distribution of the test set. The second level representation of the pyramid involves knowing the partial distribution of the test set in advance and adjusting the partial distribution of the train set to achieve a certain degree of consistency. The first level of the pyramid represents the most ideal state, where the estimated output of the network is precisely the same as the gray scale of the ground truth.

The imaging quality of the test set for each level above is higher than that of the next level (however, it is not ruled out that there may be exceptions in certain individual cases). In practice, the first level is an ideal process, and the second level requires prior knowledge of the distribution or partial distribution of the test set, which may not necessarily be satisfied in the actual process. It is often the third and fourth levels that are actually implemented. Achieving the third level is a commendable outcome. The allocation method above, Raw-1, corresponds to the fourth level.

So, we propose a new sample determination method, the CC-building method, situated at the third level of the pyramid. The principle of this method is to select a combination of samples with a smaller total correlation coefficient when the number of samples is fixed; that is, to make a combination with less “similar” samples. This method can achieve relatively good imaging results for various distribution situations without knowledge of the test set distribution. The pseudo code of the CC-building method is as Algorithm 1.
**Algorithm 1:** CC-building Method
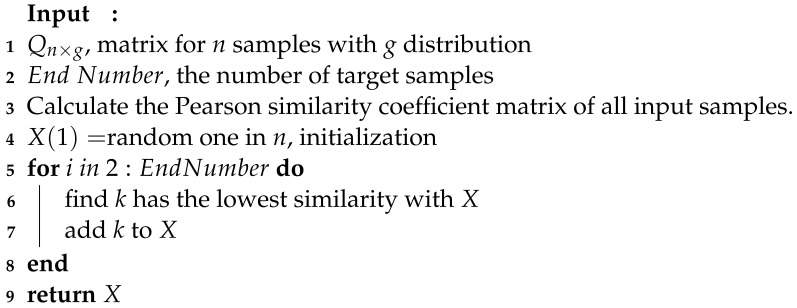


**Figure 8:**
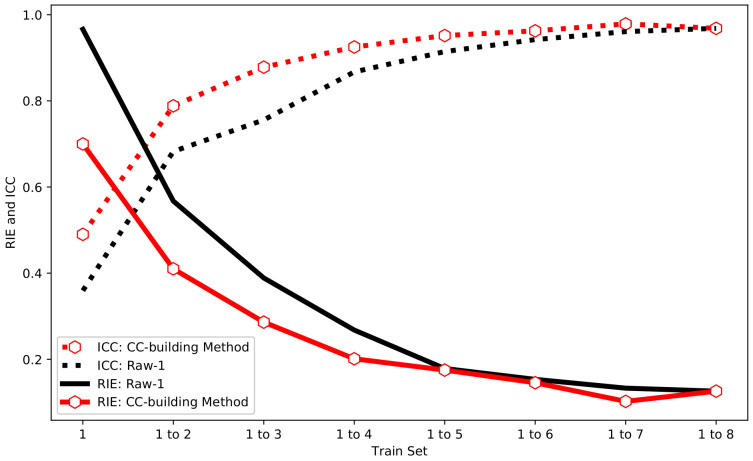
The RIE and CC of Raw-1 and CC-building method in simulation.

Compared with Raw-1, the CC-building method is used to select the same number of training sets to participate in the training. The final test results are shown in Figure 8. It can be seen from Figure 8 that the RIE and ICC of the CC-building method have the same trend as Raw-1. Besides, it can be found that the CC-building method requires fewer samples to achieve the same image reconstruction effect compared to Raw-1. This characteristic becomes more pronounced with fewer samples involved in training.

**Figure 9:**
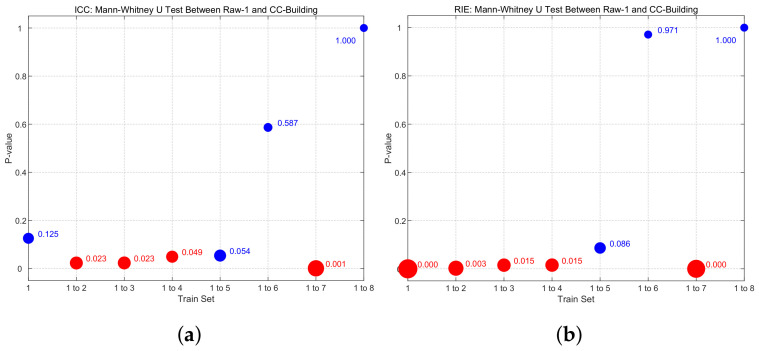
Mann–Whitney U test between RAW-1 and CC-building. (**a**) ICC: Mann–Whitney U test between RAW-1 and CC-building. (**b**) RIE: Mann–Whitney U test between RAW-1 and CC-building.

The Mann–Whitney U test is further employed to compare the statistical differences between the Raw-1 and CC-building method. In Figure 9, the size of each point represents the *p*-value between Raw-1 and CC-building, with smaller *p*-values corresponding to larger points. Points are colored red for p<0.05, indicating a statistically significant difference between the two. Conversely, points are colored blue for p⩾0.05, signifying the absence of a statistically significant difference. As shown in Figure 9, when the train set = 1 to 2, 1 to 3, 1 to 4, and 1 to 7, p<0.05. This indicates that, under this situation, the improvement in image reconstruction quality by the CC-building method relative to Raw-1 is statistically significant. This also demonstrates that optimizing the sample set has a significant improvement when the sample size is small to moderate. Considering the finer divisions in practical scenarios, optimizing the sample set should be effective over a considerably wide range of sample sizes.

Increasing the number of samples will require more time and resources to generate samples. The increase in the sample size during network training will also consume additional time and computational power. Optimizing the sample set in advance during the sample determination phase can easily and effectively improve the quality of image reconstruction.

**Figure 10:**
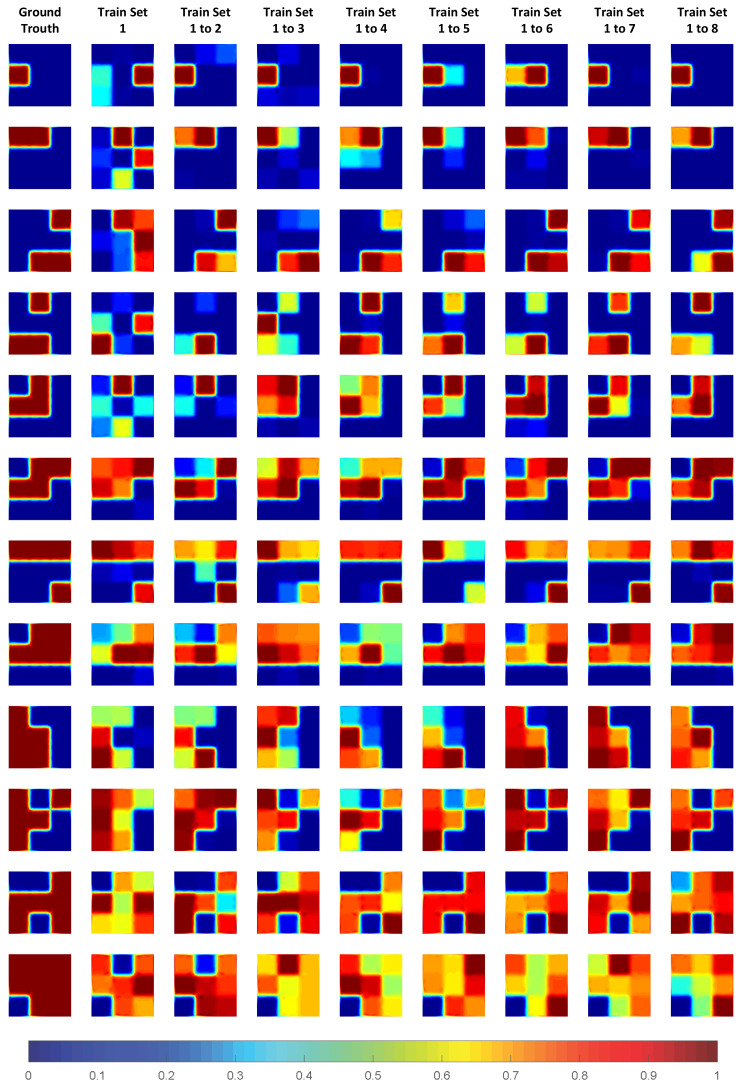
Reconstructed images of simulation.

The specific image reconstruction results of the test set are shown in Figure 10. In Figure 10, the far left is the ground truth, and columns 2 to 9 are train set = 1, train set = 1 to 2, ..., train set = 1 to 8. Looking at each line from left to right, we can clearly see the improvement of the reconstructed image by gradually increasing the size of the training set. This process has not been presented in other studies. When the train set = 1 or 1 to 2, the reconstructed image can only basically describe the part of the location of the object, with significant artifacts and errors. However, as the amount of training set data gradually increases, the missing part of the object is gradually filled, and the false artifacts are gradually corrected. When the train set = 1 to 4, it is sufficient to complete a pretty good image reconstruction result. Particularly, when the train set = 1 to 5, 1 to 6, and 1 to 7, the difference in the accuracy of the reconstructed image is indeed not significant. From this perspective, the guarantee of the number of samples is a necessary condition for DL-EMT.

## 4. Experimental Validation

**Figure 11:**
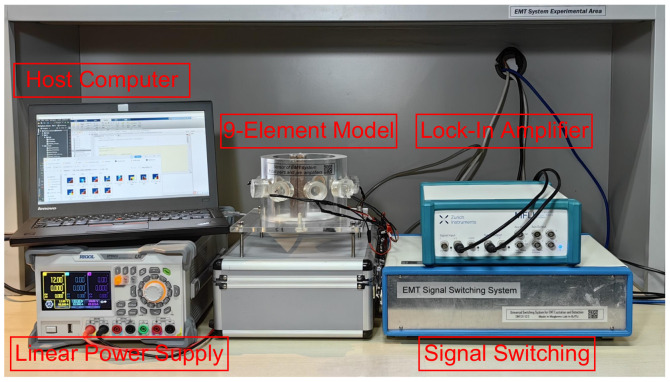
Lock-In-type EMT system.

A series of practical verification experiments were implemented in the eight-coil EMT system developed as shown in Figure 11. Lock-In Amplifier is a technology for measuring the amplitude and phase of periodic signals. It obtains the signal of frequency $\omega_{s}=2\pi f_{s}$ by comparing the signal with the reference signal.

**Figure 12:**
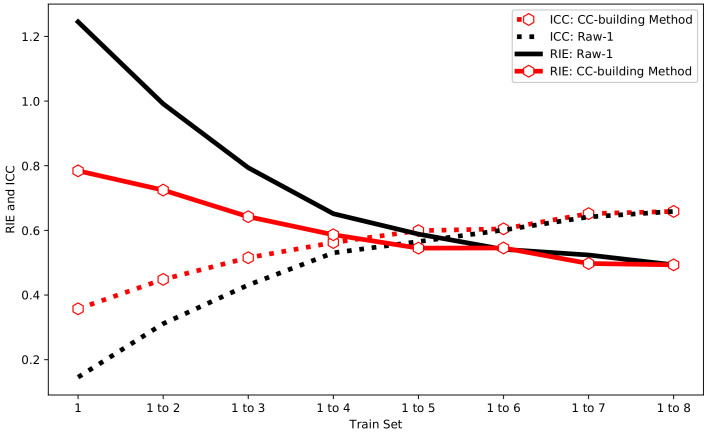
The RIE and CC of Raw-1 and CC-building method in experiment.

All samples are positioned manually, resulting in a total of 512 experimental samples being obtained. Similar to the simulation, samples with the same serial number were divided into training and testing sets. Since the conditions of simulation are relatively ideal, the experiment serves as a validation. After the experimental sample set is obtained, the process is the same as the simulation sample set, which does not need to be reiterated here. The ICC and RIE of Raw-1 and the CC-building method are shown in Figure 12.

From Figure 12, the measured samples have the same patterns as the simulated samples. With the increase in the sample size, the ICC of Raw-1 and the CC-building method gradually increases while the RIE gradually decreases. At the same sample size, the CC-building method demonstrates a significant improvement in imaging quality to Raw-1, and this advantage diminishes and eventually disappears when the sample size continues to grow. Nevertheless, compared with the simulation, the difference is that the measured overall ICC is lower and the RIE is higher. This result is also reasonable because the measured noise is larger and the environment is more complex.

**Figure 13:**
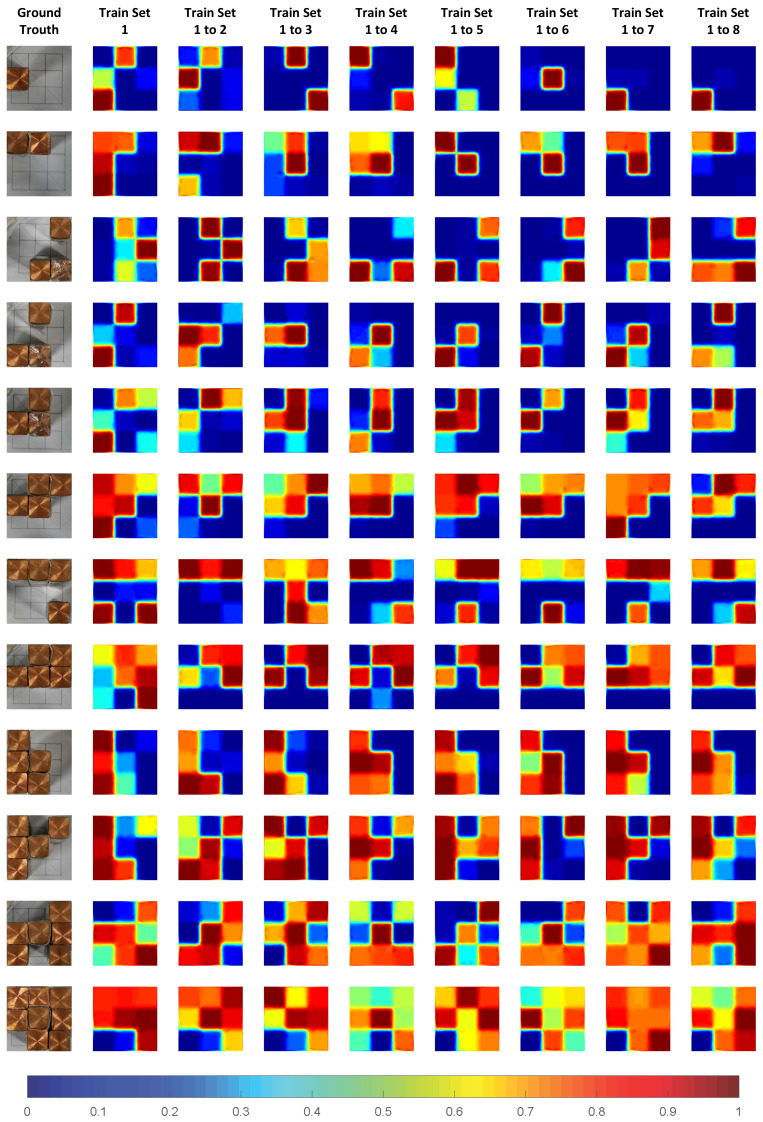
Reconstructed images of experiment.

The specific image reconstruction results of the experimental samples under the CC-building method are shown in Figure 13. It can be seen clearly from Figure 13 that the quality of the reconstructed images shows a gradual trend of improvement in the quality of the reconstructed images. However, compared to the simulation, the experiment requires more samples to ensure the effectiveness of the image reconstruction. This is also related to the error in manual placement, yet the overall trend remains unchanged.

## 5. Conclusions

This paper studies the influence of sample determination on deep learning electromagnetic tomography imaging. A basic EMT model with nine elements was established. In this model, two complete sets of 512 samples were obtained through simulation and experimental measurement. The increase in samples has a marginal effect on the same model, which increases first and then diminishes. Therefore, increasing the sample quantity after a certain point does not significantly improve the image reconstruction quality. Secondly, by optimizing the sample combination, fewer samples can achieve the same or even better image reconstruction quality compared with more samples. Based on the sample determination study, this paper proposes a sample optimization method, the CC-building method. The simulation results show that it can improve the quality of image reconstruction. This is still effective for experiments under the same conditions. We hope that this method can provide some reference for future deep learning electrical tomography research to improve the image reconstruction quality and performance.

## Data Availability

The data presented in this study are available upon reasonable request from the authors.
